# The Mongolian gerbil as an advanced model to study cone system physiology

**DOI:** 10.3389/fncel.2024.1339282

**Published:** 2024-01-25

**Authors:** Alexander Günter, Soumaya Belhadj, Mathias W. Seeliger, Regine Mühlfriedel

**Affiliations:** Division of Ocular Neurodegeneration, Centre for Ophthalmology, Institute for Ophthalmic Research, University of Tübingen, Tübingen, Germany

**Keywords:** Mongolian gerbil, visual streak, cone system, electroretinography, photoreceptors, diurnal rodents, macula, animal model

## Abstract

In this work, we introduce a diurnal rodent, the Mongolian gerbil (*Meriones unguiculatus*) (MG) as an alternative to study retinal cone system physiology and pathophysiology in mice. The cone system is of particular importance, as it provides high-acuity and color vision and its impairment in retinal disorders is thus especially disabling. Despite their nocturnal lifestyle, mice are currently the most popular animals to study cone-related diseases due to the high availability of genetically modified models. However, the potential for successful translation of any cone-related results is limited due to the substantial differences in retinal organization between mice and humans. Alternatively, there are diurnal rodents such as the MG with a higher retinal proportion of cones and a macula-like specialized region for improved visual resolution, the visual streak. The focus of this work was the evaluation of the MG’s cone system functionality using full-field electroretinography (ERG), together with a morphological assessment of its retinal/visual streak organization via angiography, optical coherence tomography (OCT), and photoreceptor immunohistochemistry. We found that rod system responses in MGs were comparable or slightly inferior to mice, while in contrast, cone system responses were much larger, more sensitive, and also faster than those in the murine counterparts, and in addition, it was possible to record sizeable ON and OFF ERG components. Morphologically, MG cone photoreceptor opsins were evenly distributed throughout the retina, while mice show a dorsoventral M- and S-opsin gradient. Additionally, each cone expressed a single opsin, in contrast to the typical co-expression of opsins in mice. Particular attention was given to the visual streak region, featuring a higher density of cones, elongated cone and rod outer segments (OSs), and an increased thickness of the inner and outer retinal layers in comparison to peripheral regions. In summary, our data render the MG a supreme model to investigate cone system physiology, pathophysiology, and to validate potential therapeutic strategies in that context.

## 1 Introduction

In the mammalian retina, rod and cone photoreceptors detect light stimuli and translate them into electrical signals that travel through the retinal network to the brain. Rods are very light sensitive and function optimally in dark-adapted (scotopic) conditions, while cones are important for photopic vision in daylight conditions and thus have a critical role in the perception of color and high-acuity cues. While all mammals possess cones, their topographical distribution across the retina varies considerably among different species (Peichl, 2005). This great variety has been attributed to the evolutionary pressure by environmental conditions, physical characteristics, and activity patterns (Schiviz et al., 2008).

In primates, the need for stereoscopic daylight vision led to the development of a specialized high-acuity region, the macula, characterized by a markedly enhanced density of cones, elongation of their outer segments (OSs) and a lack of light-blocking vascularization in the fovea (Ahnelt and Kolb, 2000; Kolb, 2005). Consequently, a higher density of bipolar and ganglion cells is found in the inner retina of this area, so that the central retina has a greater thickness than peripheral regions (Kolb, 2005). On the other hand, nocturnal species like mice (*Mus musculus*) do neither present a macula nor a specialized region with macula-like features (Huber et al., 2010), although the fraction of cones in the retina, which account for 3% of the total photoreceptor population (Carter-Dawson and LaVail, 1979), is not much different from e.g., humans. Moreover, in contrast to humans, the majority of murine cones co-express short wavelength-sensitive (SWS) and middle wavelength-sensitive (MWS) opsins, which form inversely running dorsoventral retinal gradients (Applebury et al., 2000; Wang et al., 2011).

Numerous retinal diseases affect the cone system, including neurodegenerative diseases like age-related macular degeneration (AMD) and inherited retinal degenerations, such as achromatopsia or cone dystrophies (Hadziahmetovic and Malek, 2020; Brunet et al., 2022). Even in diseases where the rods are primarily affected, a secondary cone death occurs, which is the case of retinitis pigmentosa (Brunet et al., 2022). The most disabling handicap in retinal degeneration is the loss of central vision in daylight, and therefore, there is the need for animal models with a cone system more similar to the human one. However, despite the limitations of the cone system when compared to humans, mice are currently most popular in studies of retinal disorders and the associated pathophysiology due to the availability of many genetically engineered homologous disease models.

Rodents like the *Arvicanthis niloticus*, *Arvicanthis ansorgei* and the *Octodon degus* are well described diurnal cone-rich models that have a high complement of cones in their retina with better functionality than mice, but no specialization of the vascular pattern in their retina has been described (Jacobs et al., 2003; Bobu et al., 2008; Gilmour et al., 2008; Gaillard et al., 2009; Boudard et al., 2010). Other rodents with diurnal activity are the *Psammomys obesus* and the *Meriones shawi*, which have been thoroughly studied as diabetic retinopathy models (Saïdi et al., 2011; Hammoum et al., 2017a,b, 2018; Dellaa et al., 2021). Here, we present the Mongolian gerbils (*Meriones unguiculatus*) (MGs), which are diurnal rodents that possess approx. 14% cones, about 5 times as many as mice (Govardovskii et al., 1992), along with a specialized visual streak for high-acuity vision, which enables their retina to scan the horizon for its own flock and predators in an environment of semi-deserts and steppes (Huber et al., 2010; Scheibler and Waiblinger, 2018). The presence of a retinal region analogous to the macula is particularly important to model the human situation, because even with a total amount of barely 5% of cones in our entire retina, the high concentration of these photoreceptors in the macula is the key for our very high acuity vision (Lamb, 2016). Still, the majority of our cones are located outside of this region (Robson et al., 2022) and techniques like the full-field electroretinography (ERG), which measure the functionality of the entire retina, are essential to research suitable models for cone system physiology and pathophysiology. On the other hand, to profit from the experimental tools available for mice, a phylogenetic similarity with them is very helpful e.g., for the use of commercially available antibodies.

To assess the suitability of the MG as an alternative model for human retinal cone system physiology and pathophysiology, our goals in this study were to functionally evaluate the cone system of the MG and to morphologically characterize the photoreceptors in the visual streak and adjacent regions. Further, we performed functional and morphological comparisons between the visual system of MGs and wild-type (WT) mice. As a key feature, we found that the cone system of MGs produces functionally larger, more sensitive and faster ERG responses than that of mice. In particular, light-adapted cone responses allowed for a higher flicker fusion frequency (FFF) including a prominent 30 Hz ERG, as well as distinct ON/OFF responses, which are also present in human recordings (Sustar et al., 2018; Robson et al., 2022). In addition, an increased cone density, length of OSs, and thickness of the inner retina were found in the visual streak of MGs, features similar to what is present in the human macula. In summary, our comparative data indicate that the Mongolian gerbil is a superior model to investigate cone system physiology, pathophysiology, and to validate potential therapeutic strategies.

## 2 Materials and methods

### 2.1 Experimental animals

All animal experiments and procedures performed in this study adhere to the ARVO statement for the Use of Animals in ophthalmic and Vision Research and were approved by the competent legal authority (Regierungspräsidium Tübingen, Germany). Mongolian gerbils were housed in an alternating 12-h light and dark cycle with free access to food and water. In the experiments, 9 adult MGs aged 2–3 months were used. For comparative purposes, data of adult WT mice (C57BL/6J, 1–2 months postnatally) were taken from previous recordings.

### 2.2 Electroretinography

Mongolian gerbils were dark-adapted overnight and subsequently anesthetized with a subcutaneous injection of ketamine (50 mg/kg of bodyweight) together with xylazine (2 mg/kg of bodyweight) diluted in 0.9% NaCl saline. Tropicamide drops (Pharma Stulln, Stulln, Germany) were applied to each eye for pupil dilation. All procedures were performed under dim-red light conditions. Gerbils were positioned in prone position on a heated surface for binocular recordings. Two gold-wire ring electrodes, moisturized with methylcellulose (OmniVision GmbH, Puchheim, Germany), contacted the surface of both corneas. Two short stainless-steel needles (Sei Emg s.r.l., Cittadella, Italy) were used as reference and ground electrodes, respectively. Full-field ERG recordings were performed with the Espion E^3^ console connected to a computer, a 32-bit amplifier and a Ganzfeld Bowl (Diagnosys, LLC, Lowell, MA, USA). Amplifier cutoff frequencies were 0.3 Hz (lower) and 300 Hz (upper).

Dark-adapted single flash series ranging from 1 mcd*s/m^2^ to 30 cd*s/m^2^, followed by light-adapted single flash series ranging from 10 mcd*s/m^2^ to 30 cd*s/m^2^ on a rod-saturating 30 cd/m^2^ background were performed. Each step was averaged 10–15 times. Moreover, photopic 10 cd*s/m^2^ flicker series ranging from 0.5–60 Hz were recorded. Responses were averaged 20 times for 0.5–3 Hz and 30 times for frequencies above 5 Hz. Flash duration in these single flash and flicker protocols was 4 ms. The photopic ON-OFF ERG was performed using a 1000 ms, 240 cd/m^2^, 0.11 Hz stimulus on a rod-saturating 30 cd/m^2^ background. The response was averaged 10 times. Single flash b-wave amplitudes were determined from the through of the a-wave to the peak of the b-wave and flicker response amplitudes were measured from the through to the peak of the positive deflection. Box-and-whisker plots were produced with Microsoft Excel (Microsoft Corp., Redmond, WA, USA). Mouse data for comparison had been recorded and processed in a similar way as published previously (Seeliger et al., 2005; Tanimoto et al., 2009).

### 2.3 Scanning-laser ophthalmoscopy (SLO) and optical coherence tomography (OCT)

*In vivo* imaging was performed as previously described (Fischer et al., 2009; Huber et al., 2009). In short, animals were anesthetized and pupils were dilated as described for the ERG recordings, and a 100 dpt custom-made contact lens was fitted to the cornea after application of a drop of methylcellulose. SLO imaging was performed together with OCT on the Spectralis™ HRA + OCT device using the proprietary software package Eye Explorer version 5.3.3.0 (Heidelberg Engineering, Heidelberg, Germany). This system features a super-luminescent diode at 870 nm as low coherence light source. Each two-dimensional B-Scan recorded at 30° field of view contains up to 1536 A-Scans, which are acquired at a speed of 40,000 scans per second. To perform fluorescein angiography (FLA), 75 mg/kg body weight of fluorescein were injected subcutaneously and images generated with a blue (488 nm) stimulating laser and a barrier filter at 500 nm. For indocyanine green (ICG) angiography, 50 mg/kg body weight of ICG were injected subcutaneously and images generated with an infrared (795 nm) stimulating laser and a barrier filter at 800 nm. Data were exported as 8-bit gray scale image files and processed in the CorelDRAW X5 software (Corel corporation, Ottawa, ON, Canada). Scale bars were calibrated based on OCT and SLO retina images with intraocular-injected beads of a defined diameter (Garcia Garrido et al., 2015).

### 2.4 Immunohistochemistry

Eyes were fixed in 4% Paraformaldehyde for 45 min before being immersed in 30% sucrose in phosphate buffer (pH 7.4) overnight at 4°C. They were embedded in Tissue-Tek OCT compound (Sakura Finetek Europe, Alphen aan Den Rijn, Netherlands) and frozen using dry ice. A total of 12 μm dorsoventral retinal cross-sections were collected on Superfrost glass slides (R. Langenbrinck GmbH, SuperFrost^®^ plus, Emmendingen, Germany) and stored at −20°C. The slides were dried at 37°C for 30 min and rehydrated with phosphate buffer saline (PBS) for 10 min. Afterward, they were incubated for 1 h at room temperature (RT) with a blocking solution, consisting of 5% chemiBLOCKER (Merck, Darmstadt, Germany) in 0.1% PBS Triton X-100. Next, the sections were incubated overnight at 4°C with the following antibodies: anti-SWS cone opsin (AB5407; 1:300; Merck), anti-MWS cone opsin (AB5405; 1:300; Merck), anti-MWS cone opsin (PA1-9517; 1:100; Thermo Fischer Scientific, Karlsruhe, Germany) for double staining with anti-SWS cone opsin, FITC conjugated PNA (L7381; 1:100; Sigma-Aldrich, St Louis, MO, USA) and anti-rhodopsin (ab98887; 1:500; Abcam, Berlin, Germany) diluted in blocking solution. On the next day, Slides were washed in washing solution (PBS with 2% chemiBLOCKER) and incubated for 2 h at RT with the following secondary antibodies: Goat anti-Rabbit, Alexa Fluor 568 (A11036; 1:300; Thermo Fischer Scientific), Goat anti-Chicken, Alexa Fluor 647 (A21449; 1:150; Thermo Fischer Scientific) and Goat anti-Mouse, Alexa Fluor 647 (ab150119; 1:150; Abcam) diluted in washing solution. Afterward, sections were rinsed with PBS and mounted with ROTI Mount FluorCare DAPI (Carl Roth, Karlsruhe, Germany).

### 2.5 Microscopy and image analysis

Immunohistochemical sections were imaged on a Zeiss Imager Z.2 fluorescence microscope, equipped with ApoTome 2, an Axiocam 506 mono camera and an HXP-120V fluorescent lamp (Carl Zeiss Microscopy, Oberkochen, Germany). The ZEN 3.3 (blue edition) software (Carl Zeiss Microscopy) captured z-stack images using 20x and 40x magnifications. The quantification of the rod/cone OS length, cone density and the number of outer nuclear layers (ONLs) was done by averaging measurements from at least four sections per animal. Per section, three distinct measurements were taken and averaged (Roche et al., 2016). Dorsal and ventral sections taken approx. 300 μm from the center of the visual streak were considered as peripheral retina. Figures were prepared using Photoshop CS5 (Adobe, San Jose, CA, USA). OCT reflectivity profiles between the upper ganglion cell layer and the bottom retinal pigment epithelium (RPE) layer were generated and analyzed using the ImageJ software package (NIH, Bethesda, MD, USA) as described previously (Garcia Garrido et al., 2014).

### 2.6 Statistical analysis

A one-tailed paired student’s t-test was used to assess statistical differences between quantitative data of the visual streak and the peripheral retina based on at least three individual animals. Values of *p* < 0.05 were considered to be statistically significant and labeled with an asterisk (*) in the graphs. To indicate a higher degree of statistical significance, values of *p* < 0.01 were marked with two (**) asterisks and *p* < 0.001 with three (***) asterisks. Graphical results are represented as mean ± standard deviation. The statistical analysis was performed with Microsoft Excel.

## 3 Results

### 3.1 Rod system responses are comparable between mice and Mongolian gerbils

**FIGURE 1:**
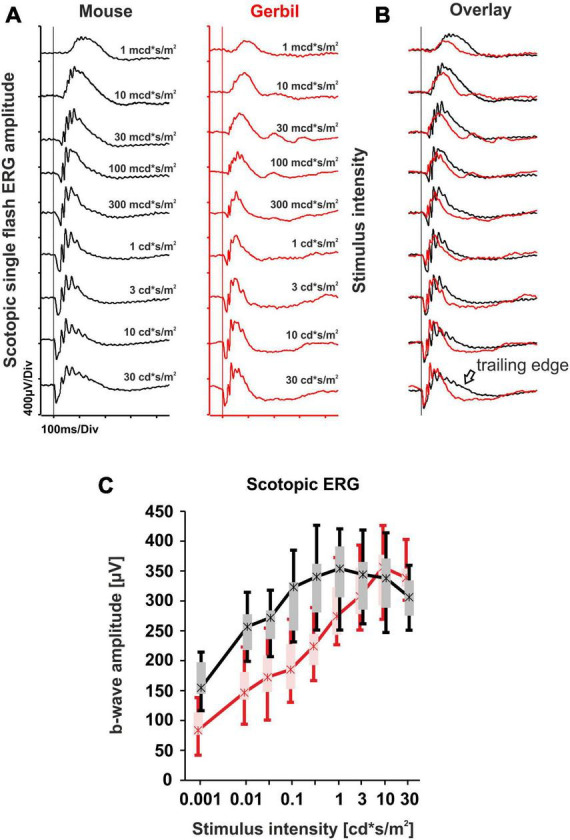
Dark-adapted single flash electroretinography (ERG) series comparison of mice and Mongolian gerbils (MGs). **(A)** Representative scotopic single flash intensity series of mice (black) and MGs (red) ranging from 1 mcd*s/m^2^ to 30 cd*s/m^2^. The vertical lines indicate the time of the light flash. **(B)** Overlay of both scotopic single flash intensity series reveals a slower response from mice at high mesopic luminances, highlighted by the presence of a trailing edge (black arrow). **(C)** Quantitative evaluation of b-wave amplitudes (box-and-whisker-plots) for the entire group of mice (*n* = 14 eyes) and MGs (*n* = 15 eyes). Boxes: 25–75% quantile range, whiskers: 5% and 95% quantiles, asterisks: median.

Rod system responses of MGs were assessed in dark-adapted (scotopic) conditions and compared to respective murine data. Initially, a dark-adapted single flash ERG series was performed (Figure 1A). The results show that in low scotopic to low mesopic light conditions (1 mcd*s/m^2^−300 mcd*s/m^2^), b-wave amplitudes in mice were relatively higher than those in MGs, while at high mesopic flash stimuli (1 cd*s/m^2^ – 30 cd*s/m^2^), the b-wave amplitudes in MGs became similar to the ones found in mice (Figure 1C). At this luminance range, the main difference observed was a faster return of the b-wave in the gerbils to the baseline, in contrast to the b-wave in mice with a more expressed trailing edge (Figure 1B). Due to differences like eye size and retinal topography, it is hard to compare absolute ERG amplitudes across species. Nevertheless, a comparison of the purely rod-driven section below 10 mcds/m^2^ and the mixed rod-cone response section at the highest intensities between MGs and mice indicates that the rod signal in the MG appears to be generally less strong in relation to the cone signal. While rod system functionality appears principally comparable, the waveform in MGs is much more shaped by cone influences than in mice (Figures 1A, C), which is reasonable given the higher fraction of cones (approx. 14% vs. 3% in mice).

### 3.2 Increased sensitivity and size of cone system responses in Mongolian gerbils as compared to mice

**FIGURE 2:**
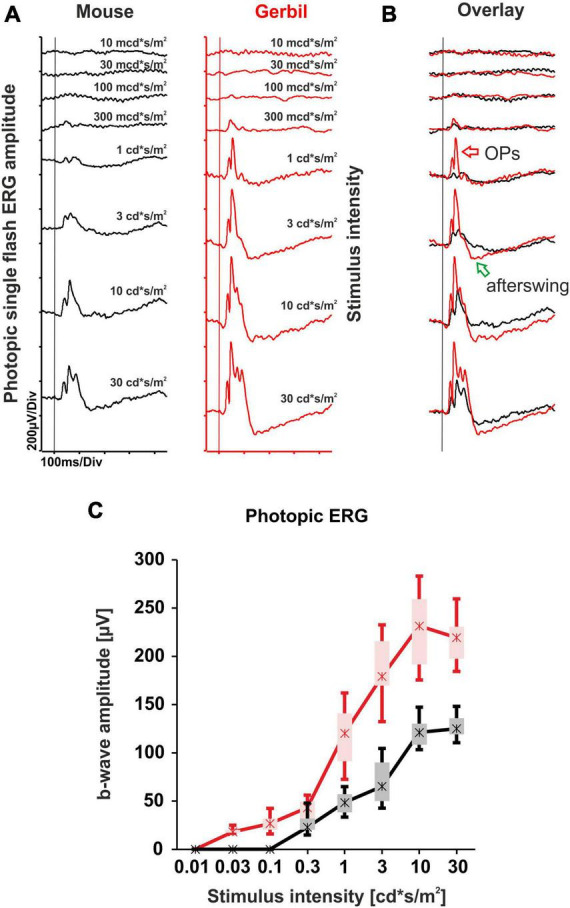
Light-adapted single flash ERG series comparison of mice and MGs. **(A)** Representative photopic single flash intensity series of mice (black) and MGs (red) ranging from 10 mcd*s/m^2^ to 30 cd*s/m^2^ on a 30 cd/m^2^ background. The vertical lines indicate the time of the light flash. **(B)** Overlay of both photopic single flash intensity series reveal larger ERG responses from MGs together with the appearance of robust Oscillatory potentials (OPs, red arrow) and negative b-wave afterswings (green arrow) at lower intensities when compared to mice. **(C)** Quantitative evaluation of b-wave amplitudes (box-and-whisker-plots) for the entire group of mice (*n* = 15 eyes) and MGs (*n* = 12 eyes). Boxes: 25–75% quantile range, whiskers: 5% and 95% quantiles, asterisks: median.

The mixed rod and cone system ERG responses presented at the higher mesopic range of the dark-adapted single flash ERG series already hinted toward a superior cone system contribution of the MG retina that was not evident in mice. To further explore performance of the cone system, we carried out a light-adapted single flash ERG series. Under these conditions, rods are saturated and the cone system predominantly contributes to the response. A comparison of the photopic ERG responses with murine records revealed that the MGs show larger b-wave amplitudes throughout the entire single flash series, with the presumed presence of a photopic hill effect between intensities of 10 and 30 cd*s/m^2^ (Figures 2A, C). The photopic hill phenomenon is also present in humans, where it is used as an additional marker for the characterization of cone system function (McCulloch et al., 2019). Moreover, the cone system of the MGs appeared more sensitive by about half a log unit, with a clearly recognizable difference to mice e.g., at the intensity of 1 cd*s/m^2^ (Figures 2A, B). Accordingly, the overlay of the ERG responses revealed that the MGs also present Oscillatory potentials (OPs) and a negative b-wave afterswing at lower intensities (Figure 2B).

### 3.3 Superior cone-specific photopic flicker ERG in Mongolian gerbils as compared to mice

**FIGURE 3:**
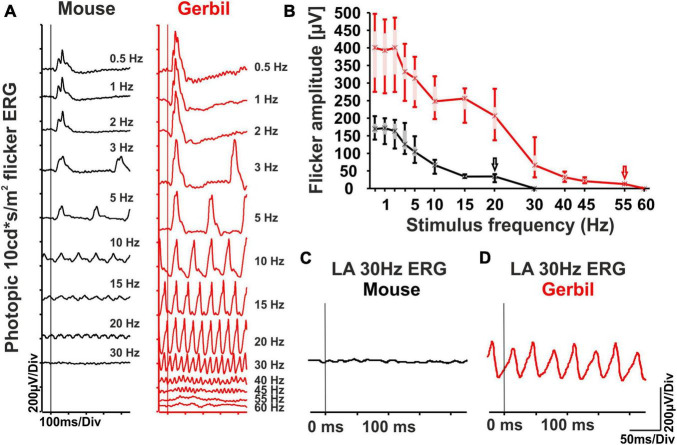
Photopic steady-state flicker ERG series in MGs and mice. **(A)** Representative 10 cd*s/m^2^ photopic flicker intensity series of mice (black) and MGs (red) ranging from 0.5 Hz to 60 Hz on a 30 cd/m^2^ background. **(B)** Quantitative evaluation of flicker ERG amplitudes (box-and-whisker-plots) for the entire group of mice (*n* = 10 eyes) and MGs (*n* = 16 eyes). Flicker responses were well recognizable until about 20 Hz in mice (black arrow) and 55 Hz in MGs (red arrow). Boxes: 25–75% quantile range, whiskers: 5% and 95% quantiles, asterisks: median. **(C,D)** Responses obtained with the photopic (light-adapted, LA) 30 Hz ERG flicker protocol issued by ISCEV. In mice, no significant response is typically found **(C)**, while the test may be successfully performed in MGs due to their superior temporal resolution **(D)**. The vertical lines indicate the start of the flash series.

As a sign of increased temporal resolution, human cone responses have a shorter duration than those in mice, which leads to a higher FFF (Tanimoto et al., 2009). To evaluate temporal resolution and the FFF in MGs, we performed light-adapted steady-state flicker ERG recordings with a 10 cd*s/m^2^ stimulus intensity and stepwise increasing flicker frequencies from 0.5 Hz to 60 Hz. While sizeable mouse retina flicker responses were limited to about 20 Hz with a FFF of 30 Hz, MG retinas were able to follow flicker until about 55 Hz with a FFF of 60 Hz (Figures 3A, B), which is very similar to what is observed in humans (Mankowska et al., 2021). Moreover, the absolute flicker amplitudes in MGs were much higher than in mice for all frequencies (Figure 3B), which would be hard to attribute to the aforementioned general species differences only. Importantly, the photopic 30 Hz flicker ERG is a standard protocol in human clinical diagnostics issued by the International Society for Clinical Electrophysiology of Vision (ISCEV) that yields robust cone system responses in healthy retinas (Robson et al., 2022). Mice, however, typically produce a flat line with this protocol due to their low FFF, while the same test in MGs leads to robust responses (Figures 3C, D). Taken together, these results further underline the superior functionality of the MG cone system, with a FFF close to human important for the performance of the diagnostic 30 Hz flicker test.

### 3.4 Excellent cone system ON and OFF responses in Mongolian gerbils widely lacking in mice

**FIGURE 4:**
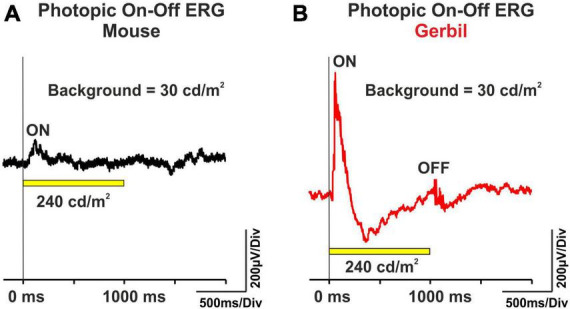
Representative photopic ON-OFF ERG curves from **(A)** mice (*n* = 8 eyes) and **(B)** MGs (*n* = 8 eyes). The ERG was performed with a 1000 ms, 240 cd/m^2^ white light stimulus (yellow bar) on a 30 cd/m^2^ background. The vertical lines indicate the onset of the light flash.

Electroretinograms in healthy human subjects contain ON and OFF components. Protocols that use long-flash stimuli on a photopic background allow for the separation of such ON and OFF responses (Sustar et al., 2018), and thus allow to detect ON- and OFF-system dysfunction in disorders affecting the inner retina. Here, we performed the photopic ON-OFF ERG protocol in MGs. While in mice, typically only small ON responses after light onset and no OFF responses after light offset were visible (Figure 4A), both substantial ON and OFF responses were recordable in MGs (Figure 4B). These results indicate that MGs have, like humans, a higher inner retinal complexity than mice.

### 3.5 The retinal topography of the Mongolian gerbil has more human-like features than that of mice

**FIGURE 5:**
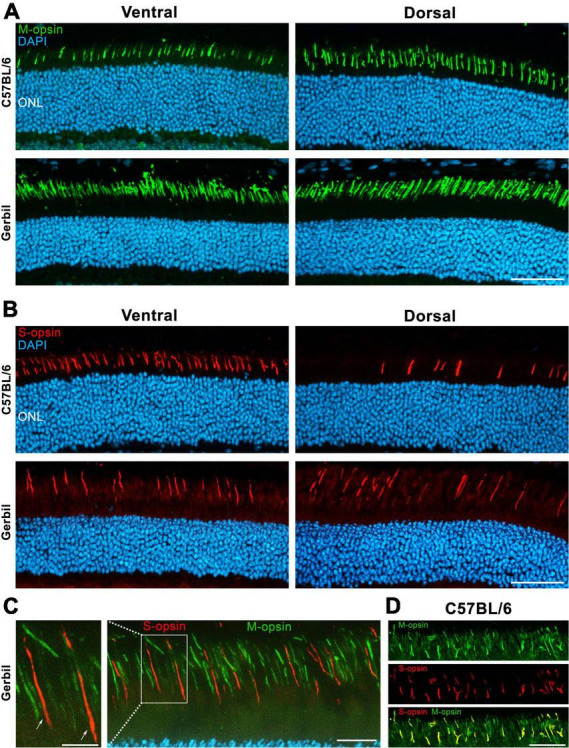
Dorsal and ventral opsin expression in the retina of a C57BL/6 mouse and a MG. **(A)** Immunohistochemical assessment indicating an increased expression of M-opsins in the dorsal in comparison to the ventral region in the retina of mice. In contrast, a generally higher density of M-cones without any dorsoventral gradient was found in MGs. DAPI was used as nuclear counterstaining **(B)** In mice, S-opsins are highly concentrated in the ventral retina, while only few of them are found in dorsal regions, presumably indicating the “true blue” cone type. Similar to murine dorsal regions, sparsely expressed S-opsins without a dorsoventral gradient are found in MGs, also resembling the “true blue” cone type. ONL, Outer nuclear layer; Scale bar = 50 μm. **(C)** A co-expression of S- and M-opsins in the outer segments (OSs) of the MG were excluded via double staining of both proteins. The left image corresponds to an enlargement of the outlined area in the right image, which is a ventral section of the MG retina. Arrows point toward two cones that express solely S-opsins. Scale bars = 5 μm (enlarged image) and 20 μm (right image). **(D)** Ventral section of the C57BL/6 mouse retina shows the co-expression of S- and M-opsins in the OSs. Scale bar = 20 μm.

A major difference between the cones of mice and humans lies in the distribution of respective opsins in their retina. In immunohistochemistry for SWS or S- and MWS or M-opsins in the retina of mice, we had found a higher content of M-opsins at the dorsal region when compared to the ventral region (Figure 5A). In contrast, S-opsins were primarily expressed in the ventral region and had a much lower density in the dorsal retina (Figure 5B). In MGs, S- and M-opsins were homogeneously distributed across the retina, with M-opsins being expressed at a much higher density than S-opsins (Figures 5A, B). Like in most other mammals, there is also an even distribution of photoreceptor opsins throughout the human retina, and with the exception of the retinal center, only a minor degree of local opsin patterning has been described (Jacobs et al., 2003; Bobu et al., 2006; Gaillard et al., 2008; Hussey et al., 2022). Moreover, while expression of S-opsins in mice is generally higher than that of M-opsins (Applebury et al., 2000), the opposite is true for MGs and humans. Further, most mammals including humans express a single opsin type per cone, whereas the majority of murine cones co-express M- and S-opsins (Figure 5D). In the retina of MGs, we found no co-expression of S- and M-opsins; each cone expressed a single opsin (Figure 5C).

### 3.6 The visual streak in the Mongolian gerbil: a specialized retinal region with reduced surface vessels and increased retinal thickness similar to the macula

**FIGURE 6:**
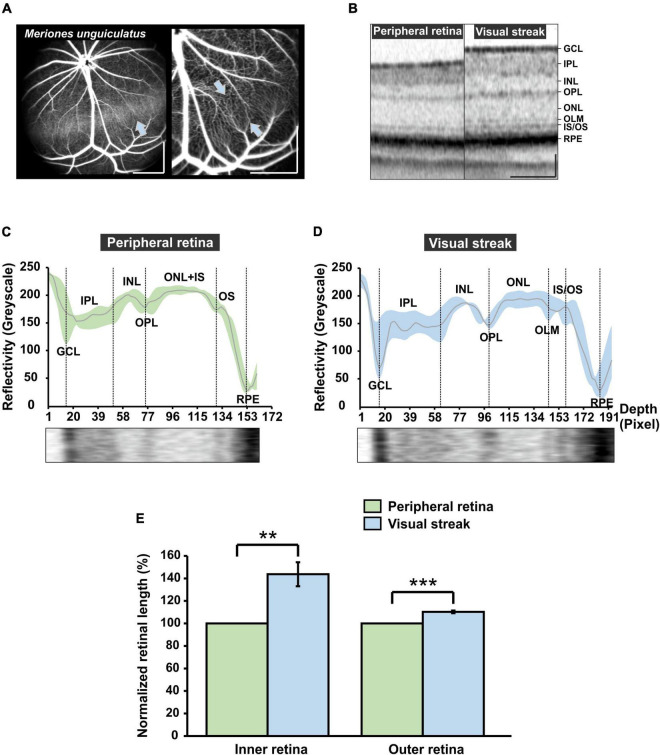
Characterization of the visual streak of the MG. **(A)** Indocyanine green (ICG) angiography (left image). The visual streak becomes visible as a horizontal band, generated by ICG in the choroidal vasculature that shines through (blue arrow). Fluorescein angiography (FLA) (right image) reveals that the streak region is vascularized mainly by thin capillaries (opposing blue arrows). Scale bar = 500 μm. **(B)** Optical coherence tomography (OCT) imaging. The visual streak imposes as a region of increased retinal thickness in comparison to the adjacent peripheral retina. Scale bar = 100 μm. **(C,D)** OCT reflectivity profile at the level of the peripheral retina [**(C)**, green] and the visual streak [**(D)**, blue]. GCL, Ganglion cell layer; IPL, Inner plexiform layer; INL, Inner nuclear layer; OPL, Outer plexiform layer; ONL, Outer nuclear layer; OLM, Outer limiting membrane; IS, Inner segment; OS, Outer segment; RPE, Retinal pigment epithelium. The x-axis indicates the retinal depth (in pixels of the image used for analysis). **(E)** Quantification of the extension of the inner retina (GCL to INL) and outer retina (OPL to RPE) in the visual streak region (*n* = 4) in comparison to the adjacent peripheral retina (*n* = 4). Visual streak data were normalized to adjacent peripheral region. Error bars indicate the standard deviation; **p < 0.01 and *** *p* < 0.001.

By taking a closer look at the retinal vasculature, ICG and fluorescein angiography images reveal a dorsally located horizontal band in the retina of the MG, the visual streak (Figure 6A). The left image (ICG angiography) provides a view of the choroidal vasculature underlying the visual streak, discernible as a lighter band in the retina (arrow in Figure 6A). In the right image, FLA was used to reveal more details of the retinal capillaries in the streak region and further illustrates the reduced number of light-blocking major surface vessels that may reduce visual performance of the visual streak. OCT imaging of the streak region revealed a distinct retinal layering with an increased retinal thickness when compared to the adjacent peripheral retina (Figure 6B). The reflectivity profile analysis of the OCT images revealed that in the visual streak, there is a more distinct separation of the ONL, the inner segment (IS) and the OS and an enhanced visibility of the outer limiting membrane (OLM) (Figure 6D), which was barely apparent in the adjacent peripheral retina (Figure 6C). Moreover, a quantification of the retinal layers revealed that the inner/outer retina in the visual streak region was 43.69% ± 10.64%/10.30% ± 1.18% thicker when compared to the adjacent peripheral retina, respectively (Figure 6E). All in all, a reduction of light-blocking surface vessels together with an increased retinal thickness in the visual streak was found in the MG, also present in the human central retina close to the fovea (Kolb, 2005).

### 3.7 The visual streak in the Mongolian gerbil: a specialized retinal region similar to the macula with longer photoreceptor OSs and a higher cone density than the peripheral retina

**FIGURE 7:**
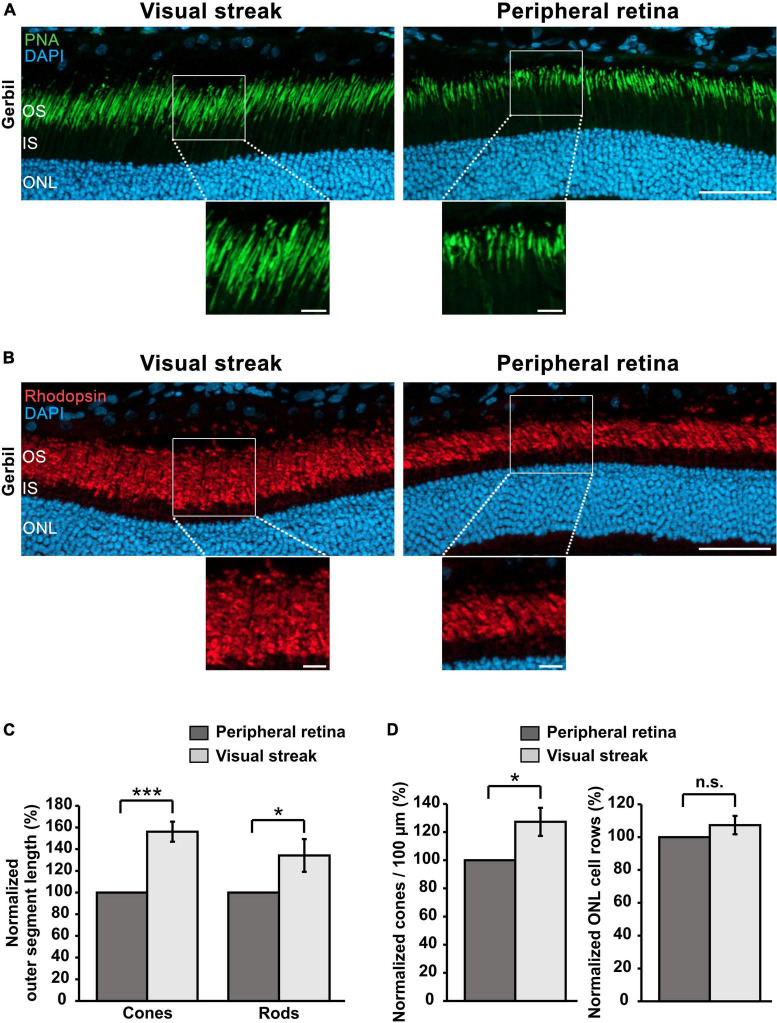
Cone/rod densities and OS lengths in the visual streak of the MG. **(A)** Immunohistochemical staining for PNA reveals the presence of longer cone OSs in the visual streak in comparison to adjacent peripheral regions. DAPI was used as nuclear counterstaining. **(B)** Immunostaining for rhodopsin indicates that rod OSs are also longer in the visual streak compared to the peripheral retina. Enlargements of the outlined areas shown below the images. ONL, Outer nuclear layer; IS, Inner segment; OS, Outer segment; Scale bars = 50 μm (upper images) and 10 μm (enlarged images). **(C)** Quantification of OS lengths of cones and rods in the visual streak (*n* = 3) in comparison to the adjacent peripheral retina (*n* = 3). **(D)** Quantification of the density of cones/100 μm and ONL rows in the visual streak (*n* = 3) in comparison to the adjacent peripheral retina (*n* = 3). Visual streak data were normalized to those of the peripheral regions. Error bars represent the standard deviation; n.s. = *p* > 0.05; *p < 0.05 and ***p < 0.001.

The retinal layers in the visual streak of the MG have unique characteristics when compared to the peripheral retina. In order to explore this further, we performed immunohistochemistry of cones and rods. Cone OSs were 56.06% ± 9.25% longer in the visual streak in comparison to the adjacent peripheral retina, which is particularly well visible in the enlargements (Figures 7A, C). A similar enlargement was also found in rods, whose OSs were 34.22% ± 15.10% longer in comparison to those in the adjacent peripheral retina (Figures 7B, C). Notably, the OSs of cones in the human fovea are also elongated, a feature believed to be related to an increased density of these photoreceptors (Hammer et al., 2008; Provis et al., 2013). In accordance with this view, we also found that the density of cones in the visual streak of the MG retina was increased by 27.29% ± 9.99% in comparison to the adjacent peripheral retina (Figure 7D). The density of rods, analyzed by comparing the number of ONL rows between these two regions, did however not differ between the visual streak and the peripheral retina (Figure 7D).

## 4 Discussion

In this study, we found that the diurnal Mongolian gerbils have considerably larger, faster and more sensitive cone system responses together with clear cone-driven ON and OFF inner retina-dominated components, both indicating that retinal function is more human-like than in mice. Morphologically, cone photoreceptor opsins in MGs were evenly distributed throughout the retina, with each cone expressing a single opsin like in human subjects, while mice in contrast show a dorsoventral M- and S-opsin gradient and a co-expression of opsins within the same cone. Additionally, the visual streak in the MG retina bears similarities with the human macula, in particular a reduced number of light-blocking surface vessels and thicker inner/outer retinas. Further, there was a higher density of cones but not rods in the outer retinal regions of the visual streak where OSs were elongated.

Importantly for the use of MGs in preclinical studies, the full-field ERG assessment indicated that the cone system functionality in the MGs is much more comparable to human recordings than that of mice. Specifically, our data show an increased sensitivity of the light-adapted single flash ERG responses together with the presence of larger b-waves amplitudes, in agreement with what has been observed in other diurnal rodents like the Unstriped soudanian grass rat (*Arvicanthis ansorgei*), the Nile grass rat (*Arvicanthis niloticus*) and the Sand rat (*Psammomys obesus*) (Gilmour et al., 2008; Boudard et al., 2010; Dellaa et al., 2017). Likewise, the high FFF in MGs in comparison to mice was also consistent with that observed in the Nile grass rat and the Common degu (*Octodon degu*s) (60 Hz vs. > 60 Hz and 48 Hz, respectively) (Jacobs et al., 2003; Gilmour et al., 2008), which is significantly closer to the > 50–90 Hz FFF found in humans (Mankowska et al., 2021) when compared to the 30 Hz FFF found in mice. Practically, this suggests that these diurnal rodents are able to better follow dynamically moving scenes. Further ERG components that have clinical relevance in humans are the OPs and the photopic negative response (PhNR), which both reflect the activity of the inner retina (Wachtmeister, 1998; Frishman et al., 2018). In the MG, OPs and the negative afterswings analogous to the PhNR were more pronounced than in mice, indicating strong inner retina functional components. Moreover, while the cone flash b-wave contains both ON and OFF components (Frishman, 2006), the underlying ON and OFF bipolar cell activity may be separated with a longer light stimulus into a b- and a d-wave (Sustar et al., 2018), which is also used in clinical diagnostics of the human retina. Our finding of a distinct d-wave of the OFF system in the MG, which is not found in mice, was also in accordance with previous reports (Yang et al., 2015) and also described in the *Arvicanthis niloticus* and the *Psammomys obesus* (Gilmour et al., 2008; Dellaa et al., 2017).

A key feature that makes diurnal rodents as the MG unique in comparison to other common laboratory species is the presence of a macula-analogue visual streak. The lack of such a specialized retinal region in many laboratory rodents has been considered a big disadvantage in terms of central retina modeling (Winkler et al., 2020). As described here, the visual streak of the MG does not only show many vascular analogies to the human macula, but also similar enhancements in retinal structure. For example, an elongation and narrowing of OSs, the latter allowing for an increased packaging density of photoreceptors (Provis et al., 2013), are important for a higher photon catch and an improved resolution of cones in the fovea of primates and humans (Ahnelt and Kolb, 2000), but are also found in the visual streak of MGs and other diurnal rodents (Gaillard et al., 2009). Moreover, the higher density of cones in the human central retina leads to an increased thickness of the inner retina due to a greater density of second-order neurons when compared to the periphery (Kolb, 2005). The same is found here in the visual streak of the MGs, indicating a similarly specialized and complex arrangement of second-order neuron networks also in the visual streak. Interestingly, to facilitate the elongation of cone OSs in the visual streak, rod OSs must also likewise increase in length. However, presumably since the rod system does not contribute to the high resolution of daylight vision (Lamb, 2016), there was no increased density of rods in the visual streak despite their elongation.

Many species have been considered so far as models to investigate cone system physiology in the context of human diseases. Non-human primates, due to their fovea, are in principle ideal models to research the human cone system. Unfortunately, high costs, limited availability of homologous inherited diseases, and ethical concerns greatly restrict their use (Verra et al., 2020). Most other mammals do commonly have a more or less expressed visual streak, and particularly in visually oriented species such as cats and dogs, there is an area centralis, an even more specialized retinal region of high cone density within the visual streak (Seeliger and Narfström, 2000; Beltran et al., 2014). Most work in these species, however, concerns veterinary research, as again economical and scientific factors like a slow reproduction rate, prolonged course of disease and challenging experimental logistics have so far limited their use as models in research on retinal degenerative diseases (Sajdak et al., 2019; Verra et al., 2020; Winkler et al., 2020). Ground squirrels are diurnal rodents phylogenetically similar to mice, but with a different retinal organization featuring a visual streak and a fraction of 85% of total cones, having been described as a model of “universal macularity” (Li, 2020). Unfortunately, they have a hibernation pattern that limits their breeding throughout the year, and also affects cone morphology (Kuwabara, 1975; Kryger et al., 1998). Tree shrews are diurnal mammals that have a retina with 95% of total cones and present a specialized region with high ganglion cell density, however there is currently an overall limited access to them along with a lack of specific antibodies for research, since they are not rodents (Müller and Peichl, 1989; Larson-Casey et al., 2020). Moreover, the extremely high fraction of cones and the lack of a distinct rod system in these species is not ideal to model human vision, as a comparable interaction of rod and cone systems is critical to understand many retinal pathologies (Wässle, 2004). In this study, we found that the functional performance of the rod system in the MG was about comparable to that in the murine counterpart, permitting a substantial interaction of rod and cone systems as desired.

Like in most species besides mice, the use of MGs in the assessment of therapeutic strategies is currently limited by the lack of disease models with defects homologous to human disorders. However, the whole-genome sequencing of the MG has already been concluded (Zorio et al., 2019), and the first successful CRISPR/Cas9-mediated gene editing in the MG has also been performed (Wang et al., 2020). It is in our view merely a question of time until the first genetically engineered MG models will become available for research on human retinal diseases. Further, important for preclinical work, the cones of MGs were shown to produce a long-term expression of target proteins following viral delivery of transgenes (Mauck et al., 2008). In conclusion, the functional and morphological characteristics described here render the MG a superior rodent model to investigate cone system physiology, pathophysiology, and potential therapeutic strategies for human diseases in that context.

## Data availability statement

The raw data supporting the conclusions of this article will be made available by the authors, without undue reservation.

## Ethics statement

The animal study was approved by the Regierungspräsidium Tübingen, Germany. The study was conducted in accordance with the local legislation and institutional requirements.

## Author contributions

AG: Conceptualization, Data curation, Formal Analysis, Investigation, Methodology, Visualization, Writing–original draft, Writing–review and editing. SB: Investigation, Writing–review and editing. MS: Conceptualization, Funding acquisition, Project administration, Resources, Supervision, Writing–review and editing. RM: Data curation, Funding acquisition, Project administration, Resources, Supervision, Writing–review and editing.
